# Genome-Wide Identification and Expression Analysis of the *14-3-3* Family Genes in *Medicago truncatula*

**DOI:** 10.3389/fpls.2016.00320

**Published:** 2016-03-22

**Authors:** Cheng Qin, Linming Cheng, Jingqin Shen, Yunhong Zhang, Huimin Cao, Dan Lu, Chenjia Shen

**Affiliations:** College of Life and Environmental Sciences, Hangzhou Normal UniversityHangzhou, China

**Keywords:** 14-3-3 gene family, *Medicago truncatula*, *Sinorhizobium meliloti* infection, nodule formation, flowering

## Abstract

The 14-3-3 gene family, which is conserved in eukaryotes, is involved in protein-protein interactions and mediates signal transduction. However, detailed investigations of the *14-3-3* gene family in *Medicago truncatula* are largely unknown. In this study, the identification and study of *M. truncatula* 14-3-3-family genes were performed based on the latest *M. truncatula* genome. In the *M. truncatula* genome, 10 14-3-3 family genes were identified, and they can be grouped into ε and non-ε groups. An exon-intron analysis showed that the gene structures are conserved in the same group. The protein structure analysis showed that 14-3-3 proteins in *M. truncatula* are composed of nine typical antiparallel α-helices. The expression patterns of *Mt14-3-3* genes indicated that they are expressed in all tissues. Furthermore, the gene expression levels of *Mt14-3-3* under hormone treatment and *Sinorhizobium meliloti* infection showed that the *Mt14-3-3* genes were involve in nodule formation. Our findings lay a solid foundation for further functional studies of 14-3-3 in *M. truncatula*.

## Introduction

The 14-3-3s form a family that is universally expressed in eukaryotes. They are involved in protein-protein interaction and mediate signal transduction, which requires specific phosphorylated motifs. Structural studies showed that the 14-3-3 proteins consist of nine typical antiparallel α-helices (Jones et al., 1995). They often form homodimers or heterodimers, and each 14-3-3 protein in the dimer is able to interact with a different protein (Wilker et al., 2005). This property allows them to bring two different proteins together as a protein complex. Thus, 14-3-3s play important roles in several biological pathways, such as protein movement, protein interactions and protein stability (Sehnke et al., 2002; Aitken, 2006; Yang et al., 2006; Paul et al., 2012). 14-3-3 proteins are phosphotheonine/phosphoserine binding proteins that are ubiquitous in eukaryotes (Ferl, 1996). The target binding sites are RSXpSXP, RSXXpSXP, and YpT (Sehnke et al., 2002).

In recent years, the 14-3-3 family was found in several plants, including *Arabidopsis*, rice, tomato, soybean, and populus (Rosenquist et al., 2001; Jin et al., 2005; Xu and Shi, 2006; Li and Dhaubhadel, 2011; Tian et al., 2015). In plants, 14-3-3-family genes also play essential roles in signal transduction during environmental stresses (Yan et al., 2004; Aksamit et al., 2005; Xu and Shi, 2006). Additionally, many studies showed that 14-3-3 have essential roles in plant biotic stresses. In soybean, 14-3-3s play an important role in mature nodule development. When the expression level of the *14-3-3* homolog *SGF14c/SGF14l* was reduced, the nodule development and maturation in soybean was reduced. The cytoplasm of the nodule primordia cells was degraded during the early nodule development when the SGF14c/SGF14l expression level was reduced (Radwan et al., 2012).

In order to study the function of 14-3-3 proteins in plants, several high-throughput proteomic studies have been carried out to search potential 14-3-3 interactors (Chang et al., 2009; Paul et al., 2009; Jaspert et al., 2011), it was found that 14-3-3 may involve in many progresses, such as in primary metabolism, transcription, ion transport, hormonal signaling, pathogen and wounding response and so on (Chang et al., 2009; Paul et al., 2009; Jaspert et al., 2011). Furthermore, it was also found that fundamental 14-3-3 interaction complexes are highly conserved across eukaryotes (Paul et al., 2009).

The 14-3-3 proteins can interact with transcriptional regulators in plant hormone responses, such as brassinosteroid (BR), abscisic acid (ABA), and methyl jasmonate (Me-JA) (Gampala et al., 2007; Schoonheim et al., 2007; Wang et al., 2011; Tian et al., 2015). For example, the two *14-3-3* genes in *Arabidopsis, AtGRF6* and *AtGRF8*, can negatively regulate the nuclear accumulation of an important transcription factor in BR signal transduction, BZR1 (Gampala et al., 2007; Wang et al., 2011). But the functions of the 14-3-3s in other hormone stress responses in *M. truncatula*, like auxin, salicylic acid (SA), JA, and ABA, which were thought to be involved in rhizobium infection and nodulation organogenesis (Yang et al., 2015), are still not understood. *M. truncatula* is a model legume plant that can interact with rhizobia to develop nodules, which are used to fix the nitrogen (van Noorden et al., 2007; Nallu et al., 2013). No matter how much information has been reported on 14-3-3s in other plants, there is little information on the 14-3-3s in *M. truncatula*. To study the 14-3-3 family in *M. truncatula*, we identified 10 family members and studied their gene and protein structures, performed phylogenetic and promoter analyses, and analyzed their expression patterns in different tissues, as well as after hormone treatments and *Sinorhizobium meliloti* infection. These results provide useful information for further functional studies of the 14-3-3s in *M. truncatula*.

## Materials and methods

### Plant materials and growth conditions

The plant used in this experiment was *M. truncatula* (Jemalong) A17. Seeds were first treated with sulfuric acid and then allowed to germinate on 1% agar plates after being washed with sterilized water. Then, the seedlings were grown in BNM solution (Engstrom et al., 2002). Seedlings were grown at 22°C in a growth chamber with a 16/8 h (day/night) photoperiod. Then, 14-day-old seedlings were used for hormone treatments. The concentrations of each hormone treatment were as follows: indole-3-acetic acid (IAA) at 1 μM; SA at 0.5 mM; Me-JA at 100 μM, and ABA at 100 μM. Roots of the plants were collected for RNA isolation. Three independent biological repeats were used in this experiment.

### Identification of *Mt14-3-3* genes

*M. truncatula* 14-3-3 proteins were identified in the phytozome v10 database (http://phytozome.jgi.doe.gov) by keyword search (using the keyword 14-3-3). All of the protein domains of putative 14-3-3 genes were examined using InterPro (http://www.ebi.ac.uk/).

### Phylogenetic tree and gene structure analysis

Sequences of *Mt14-3-3* genes were obtained from the *M. truncatula* database. The exon/intron organization of the *Mt14-3-3* genes were identified at the GSDS website (http://gsds2.cbi.pku.edu.cn). The phylogenetic tree of the *Mt14-3-3* family was constructed by MEGA 5.1, and the Neighbor-joining was with 1,000 bootstraps. Genes with 99% bootstrap values were determined to be sister-gene pairs.

### *Cis*-element analysis

The genomic sequence 2 kb upstream of the *Mt14-3-3* genes was downloaded from the *M. truncatula* database. Nine putative *Mt14-3-3 cis*-elements were investigated in this study. Furthermore, the results were confirmed by software PLACE (http://www.dna.affrc.go.jp/PLACE/).

### Rhizobia infection

The 14-day-old seedlings were inoculated with *S. meliloti* strain (strain 1021, ATCC® Number: 51124, OD_600_ = 0.1) in the BNM medium (10 g/L tryptone, 5 g/L yeast extract, 10 g/L NaCl, 2.6 mM MgSO_4_, 2.6 mM CaCl_2_) for the rhizobial infection experiment, and the seedlings in the nitrogen-free BNM medium only containing 10 mM MgSO_4_ were used as control. Roots of the control and inoculated plants were collected at 0, 12, 24, 48, and 72 h after treatment. Then, all of the samples were used to extract RNA.

### RNA isolation and quantitative real-time PCR

Total RNA was extracted using RNeasy plant mini kits (Qiagen, Hilden, Germany). cDNA was synthesized using a PrimeScript cDNA Synthesis Kit (Takara, Dalian, China). Quantitative RT-PCR was performed on a CFX96 (Bio-Rad) using the iQ SYBR Green Supermix, and the primers used are listed in Table S1. The amplification program was 95°C for 10 s and 55°C for 30 s. For each biological sample, quantitative RT-PCR was repeated three times. The relative expression level of each gene was calculated using formula 2^−ΔΔCt^ and normalized to *MtActin* mRNA.

### Statistical analysis

The significances of the differences between two groups of data were calculated using Student's *t*-test. Significant differences were indicated with an asterisks (^*^), *P* < 0.05.

## Results

### Genome-wide identification of *14-3-3* family members in *M. truncatula*

After analyzing the phytozome website, 11 potential *14-3-3*-family genes were used as query against the *M. truncatula* genome. Of these, 10 were selected for further analyses, and one sequence (Medtr0137s0020) was excluded because it had a 99% ORF identity with another candidate *14-3-3* gene (Medtr3g014060, *Mt14-3-3d*; Figure S1). Based on their chromosomal positions, they were named *Mt14-3-3a* to *Mt14-3-3j.* All of the basic information on these 10 genes is provided in Table 1. The deduced Mt14-3-3 proteins contained 249 (Mt14-3-3d) to 285 (Mt14-3-3g) amino acid residues, and their molecular masses ranged from 28.4 kDa (Mt14-3-3f) to 32.9 kDa (Mt14-3-3g). The isoelectric points were from 4.58 (Mt14-3-3f) to 4.95 (Mt14-3-3g), which were similar to the values of 14-3-3 families in other plants (Rosenquist et al., 2001; Jin et al., 2005; Xu and Shi, 2006; Li and Dhaubhadel, 2011; Tian et al., 2015).

**Table 1:** **14-3-3 gene family in *Medicago truncatula***.

						**Deduced polypeptide**		
**Gene**	**Locus ID**	**ORF length (bp)**	**No. of introns**	**Chromosome**	**Chr. Location**	**Length (aa)**	**Mol wt (kDa)**	**pI**
*Mt14-3-3a*	Medtr2g015980	777	5	Chr2	4801805–4805858	258	29.2	4.65
*Mt14-3-3b*	Medtr2g076960	792	5	Chr2	32198695–32203902	263	30.1	4.67
*Mt14-3-3c*	Medtr2g091225	786	5	Chr2	39355216–39360728	261	29.5	4.67
*Mt14-3-3d*	Medtr3g014060	750	3	Chr3	3896608–3899205	249	28.9	4.94
*Mt14-3-3e*	Medtr3g099380	783	3	Chr3	45553554–45556835	260	29.3	4.62
*Mt14-3-3f*	Medtr3g100620	759	3	Chr3	46283001–46285737	252	28.4	4.58
*Mt14-3-3g*	Medtr4g083060	858	6	Chr4	32312133–32315511	285	32.9	4.95
*Mt14-3-3h*	Medtr5g044160	780	5	Chr5	19413443–19417374	259	29.5	4.82
*Mt14-3-3i*	Medtr5g064580	783	3	Chr5	27124640–27127449	260	29.2	4.60
*Mt14-3-3j*	Medtr8g086270	780	4	Chr8	35774133–35777967	259	29.4	4.74

### Structural analysis of *Mt14-3-3* genes

The 10 *14-3-3* genes in *M. truncatula* are located on five different chromosomes. There are three Mt14-3-3 genes located on chromosomes 2 and 3, two on chromosome 5 and only one *Mt14-3-3* gene on chromosomes 4 and 8 (Table 1).

**Figure 1:**
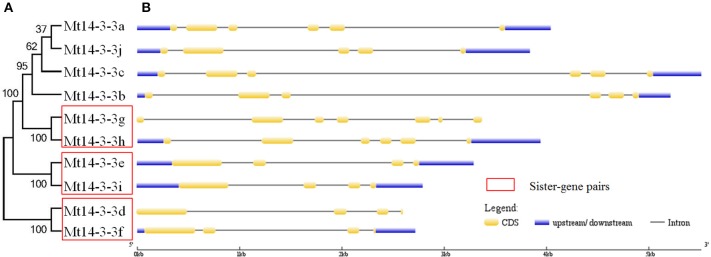
**Phylogenetic tree and structures of *Mt14-3-3* genes**. **(A)** Phylogenetic tree of *Mt14-3-3* genes. Red boxes indicate the sister-pair genes. **(B)** Exon-intron structure of the *Mt14-3-3s*. Blue boxes indicate the untranslated regions; yellow boxes indicate the exons; and gray lines indicate the introns.

To determine the gene structures of *Mt14-3-3* family members, the mRNA and the genomic DNA sequences were downloaded from the phytozome database. By comparing the mRNA and the genomic DNA sequences, the gene structures were obtained (Figure 1). The gene structure of the *Mt14-3-3* family members was found to be conserved, sharing an intron-exon pattern, especially within the same phylogenetic group (Figure 1). Furthermore, in this study, three sister-gene pairs, *Mt14-3-3g*/*Mt14-3-3h, Mt14-3-3e*/*Mt14-3-3i*, and *Mt14-3-3d*/*Mt14-3-3f*, were identified (Figure 1).

### Phylogenetic and protein structure analysis of 14-3-3s in *M. truncatula*

**Figure 2:**
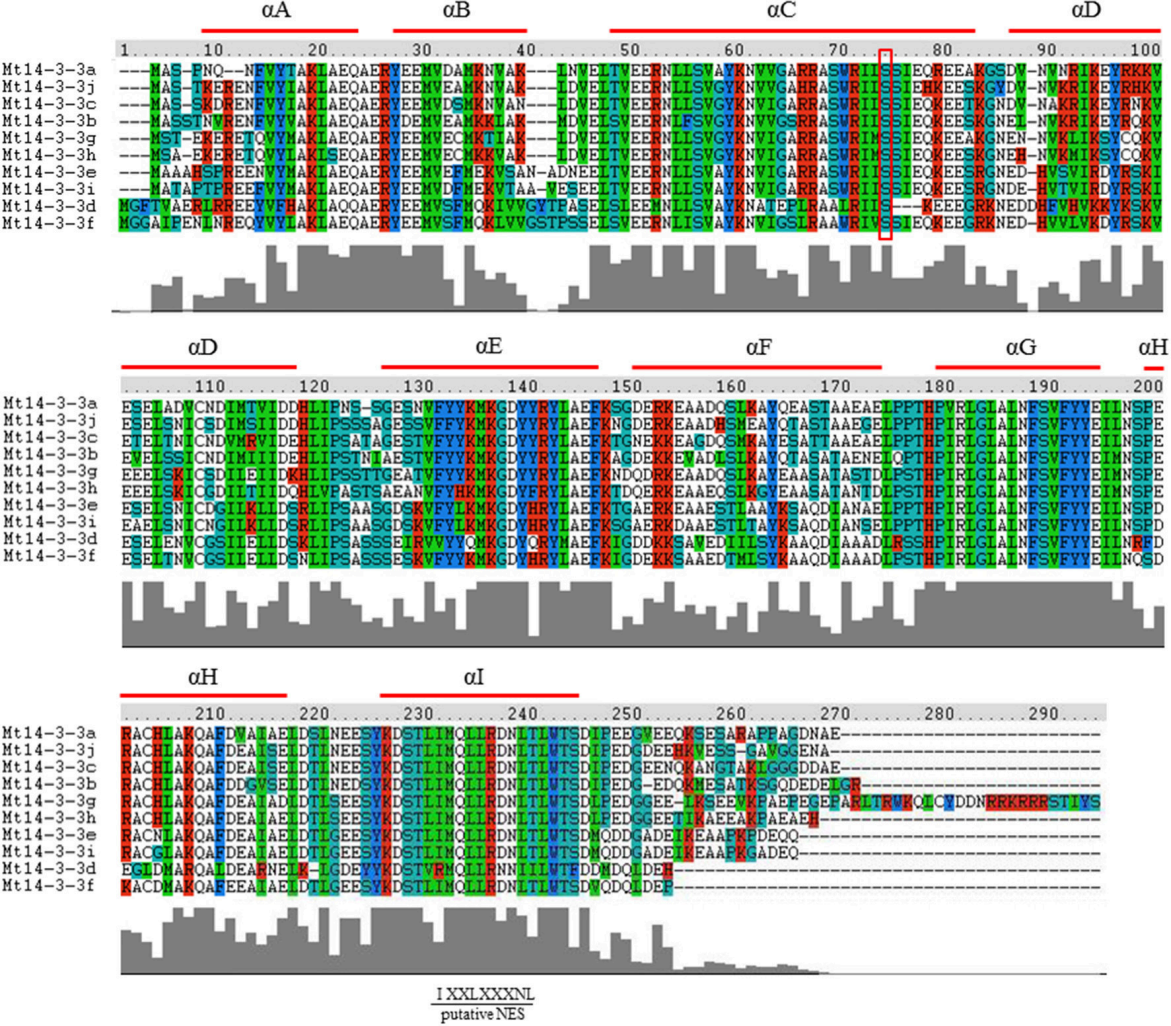
**Protein structure analysis of the Mt14-3-3 family**. Alignment of *M. truncatula* 14-3-3 proteins using the ClustalW program. Domains A–I of the Mt14-3-3 proteins are shown by red lines. Colors indicate identical amino acid residues. Putative NES indicates a putative nuclear export signal located in helix I. The red box indicates the conserved phosphorylation sites in Mt14-3-3 proteins.

The 14-3-3 family is thought to be a conserved family in plants, and the typical 14-3-3 protein contains nine antiparallel α-helices. To study the protein structure of the 14-3-3 family members in *M. truncatula*, the deduced amino acid sequences from the 10 full-length Mt14-3-3 sequences were aligned (Figure 2), and the 14-3-3 proteins in *M. truncatula* were all composed of nine antiparallel α-helices (Figure 2). Helices A through D are thought to be involved in dimerization, and helices E through I form a domain that functions in binding target proteins. Helices A, B and F showed little similarity, while helices C, E, G, and I shared an extensive identity. The conservation of the Mt14-3-3 proteins structure indicated that these proteins likely have functions that are similar to other 14-3-3s. Besides, it was showed that the N and C-terminus of Mt14-3-3 proteins being divergent, especially the amino acids in the C-terminus, which are important in calcium binding and dimer formation. This was just like the 14-3-3 proteins in other plants, such as *Arabidopsis* (Chung et al., 1999). The divergent termini may relate to functional divergence, which could give each protein its specific function by binding a range of possible target proteins.

14-3-3 can be phosphorylated by CDPK (calcium-dependent protein kinase; Swatek et al., 2014), it demonstrated that there are seven phosphorylation sites of 14-3-3 phosphorylation by CDPKs on *Arabidopsis* isoforms χ and ε, but only one site was conserved, which was also conserved in *M. truncatula* (Figure 2).

**Figure 3:**
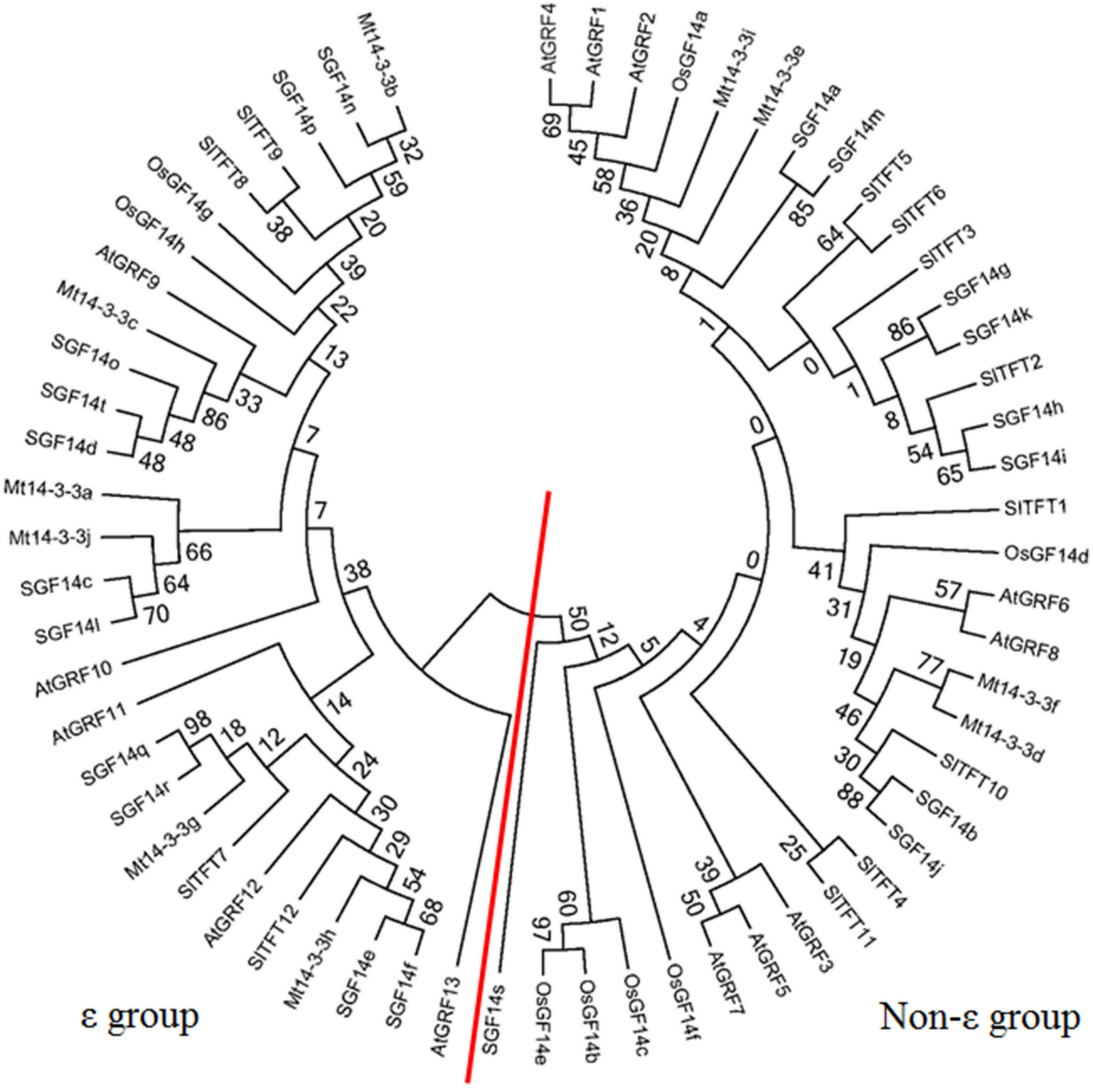
**Phylogenetic tree of *14-3-3* homologs in plants**. Phylogenetic trees of *14-3-3-*family genes in five plants constructed using the MEGA5.1 program. The red line separates the ε and non-ε groups.

To investigate the phylogenetic relationships of 14-3-3s in plants, a phylogenetic tree was constructed using the Neighbor-joining method. The phylogenetic tree included five plants of *M. truncatula, Arabidopsis*, rice, tomato and soybean. The gene names and locus IDs of these 14-3-3 genes are listed in Table S2. The 14-3-3 gene family was conserved in these plants, and they could be classified into ε and non-ε groups (Figure 3).

### *Cis*-elements in the promoters of *Mt14-3-3s*

In the promotors of genes, specific *cis*-elements motifs exist. They can be bound by transcription factors and involved in gene expression regulation, therefore, determining their presence in promoters can aid in understanding gene regulation. We searched for nine known *cis*-elements, dehydration and cold response (DRE/CRT, RCCGAC), ABA-responsive element (ABRE, C/TACGTGG/T), ARFs binding site AuxRE (AuxRE, TGTCTC), SA-responsive promoter element (SARE, TGACG), environmental signal response element (G-box, CACGTG), WRKY binding site (W-box, TTGACC/T), CAMTA binding site (CG-box, VCGCGB), phosphate starvation-responsive element (P1BS, GNATATNC), and sulfur-responsive element (SURE, GAGAC) in the promoter region of the 10 *Mt14-3-3* genes (Feng et al., 2015). These nine *cis*-elements were all located in the promoter regions of the 10 *Mt14-3-3s* (Table 2). SARE and W-box *cis*-elements existed in the upstream flanking regions of six *Mt14-3-3* genes, SARE *cis*-elements existed in *Mt14-3-3b, Mt14-3-3c, Mt14-3-3d, Mt14-3-3f*, *Mt14-3-3g*, and *Mt14-3-3i*, while W-box *cis*-elements existed in *Mt14-3-3b, Mt14-3-3c, Mt14-3-3d, Mt14-3-3h, Mt14-3-3i*, and *Mt14-3-3j*. Two kinds of *cis*-elements, ABRE and SURE, existed in the promoter region of four *Mt14-3-3s*. ABRE existed in *Mt14-3-3a, Mt14-3-3c, Mt14-3-3f*, and *Mt14-3-3g*. SURE existed in *Mt14-3-3e, Mt14-3-3f*, *Mt14-3-3g*, and *Mt14-3-3i*. AuxRE, G-box, CG-box, and P1BS existed in the promoters of only two *Mt14-3-3* genes, AuxRE was located in *Mt14-3-3g* and *Mt14-3-3h*, G-boxes were located in *Mt14-3-3a* and *Mt14-3-3c*, CG-box were located in *Mt14-3-3b* and *Mt14-3-3g*, and P1BS were located in *Mt14-3-3a* and *Mt14-3-3e*. Meanwhile, DRE/CRT *cis*-element did not exist in the promoter regions of the *Mt14-3-3* genes, except for *Mt14-3-3f*. These results indicated that Mt14-3-3 family was involved in many stress responses.

**Table 2:** **Numbers of stress-related *cis*-elements in the upstream 2 kb regions of *Mt14-3-3* genes**.

	**DRE/CRT**	**ABRE**	**AuxRE**	**SARE**	**G-box**	**W-box**	**CG-box**	**P1BS**	**SURE**
*Mt14-3-3a*	0	1	0	0	1	0	0	1	0
*Mt14-3-3b*	0	0	0	3	0	1	1	0	0
*Mt14-3-3c*	0	1	0	1	1	1	0	0	0
*Mt14-3-3d*	0	0	0	2	0	2	0	0	0
*Mt14-3-3e*	0	0	0	0	0	0	0	1	2
*Mt14-3-3f*	2	1	0	1	0	0	0	0	1
*Mt14-3-3g*	0	1	1	3	0	0	2	0	1
*Mt14-3-3h*	0	0	1	0	0	1	0	0	0
*Mt14-3-3i*	0	0	0	1	0	1	0	0	4
*Mt14-3-3j*	0	0	0	0	0	1	0	0	0

### Expression pattern of *Mt14-3-3* genes

**Figure 4:**
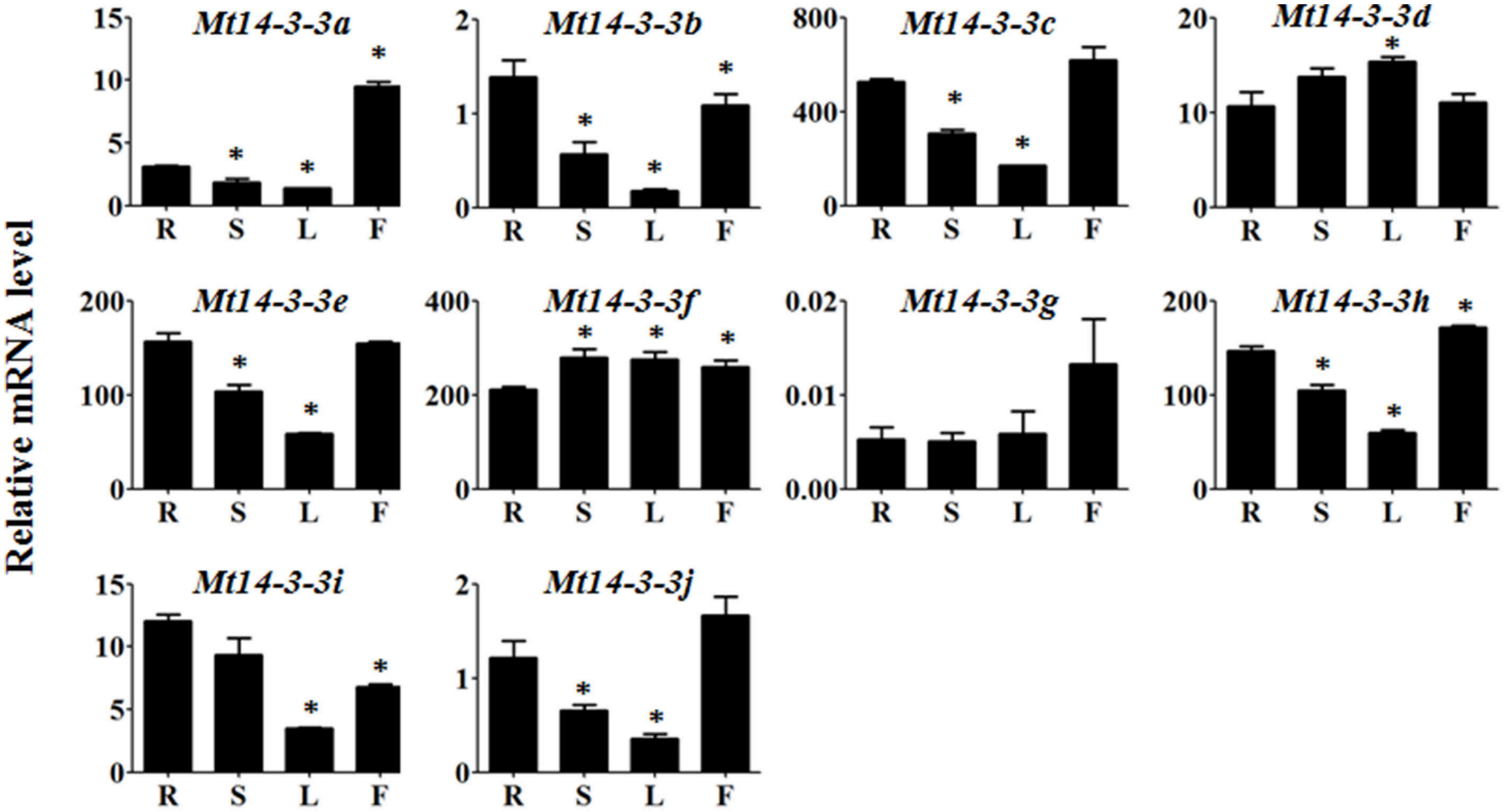
**Expression patterns of *Mt14-3-3* genes**. Expression patterns of the *Mt14-3-3* genes were analyzed in different tissues: R, root; S, shoot; L, leaf; and F, flower. The data are from three independent biological experiments, and error bars indicate standard deviations. The significant differences in expression level were calculated using Student's *t* test, and indicated with an asterisks (^*^), *P* < 0.05.

To investigate the possible roles of the *Mt14-3-3* genes, the expression pattern of these genes were determined in different tissues, including roots, stems, leaves and flowers. Quantitative real-time PCR results indicated that all 10 of the *Mt14-3-3* genes were expressed in almost every tissues of the plant (Figure 4). The expression levels of 8 *Mt14-3-3* genes were at their lowest in leaves compared with in other tissues, except for *Mt14-3-3d* and *Mt14-3-3f*, which were expressed almost equally in all of the tissues.

As the expression data for most genes in *M. truncatula* can be accessed at the MtGEA website (http://mtgea.noble.org; Benedito et al., 2008), we looked at the expression levels of the *Mt14-3-3* genes in different tissues. The probeset IDs of the *Mt14-3-3* genes are listed in Table S3. It showed almost the same expression pattern as the *Mt14-3-3s* (Figure S2).

### Expression levels of *Mt14-3-3* genes after *S. meliloti* infection

The 14-3-3s are involved in many physiological processes through the binding and regulating of important proteins. Additionally, the 14-3-3s play an essential role in the early developmental stages of nodules in soybean (Radwan et al., 2012).

**Figure 5:**
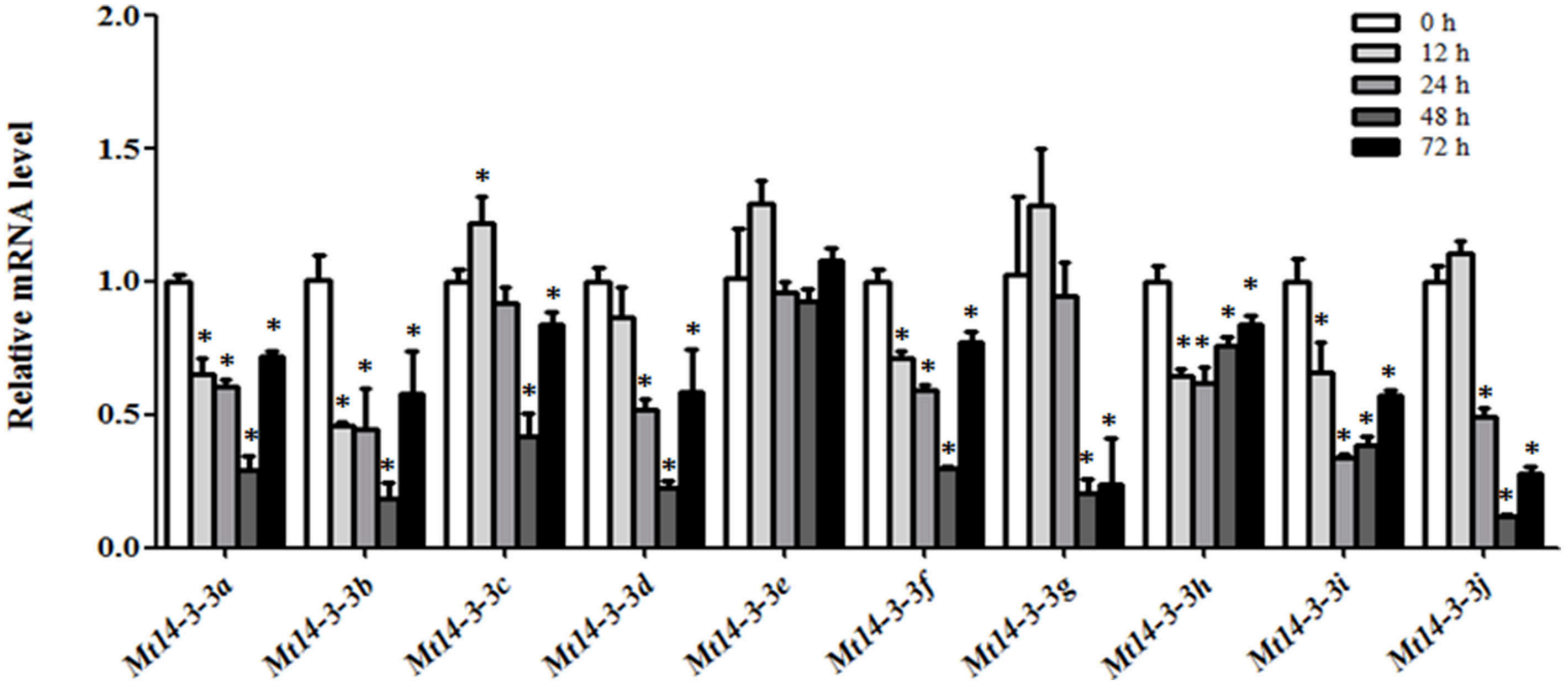
**Expression of *Mt14-3-3* genes after *S. meliloti* infection**. The expression levels of *Mt14-3-3* genes were tested at different time points (12, 24, 48, and72 hpi) after being infected with *S. meliloti*. The data are from three independent biological experiments, and error bars indicate standard deviations. The significant differences between data were calculated using Student's *t*-test, and indicated with an asterisks (^*^), *P* < 0.05.

Because we were also interested in *Mt14-3-3* gene roles in nodule formation in *M. truncatula*, we detected their expression levels after infection by *S. meliloti.* As determined by qRT-PCR, the expression levels of almost all of the *Mt14-3-3* genes showed a drastic decline in the roots during the first 48 h of *S. meliloti* infection, reaching their lowest expression levels 48 h after infection. Then, they increased 72 h after infection, except for *Mt14-3-3e*, which showed no obvious changes after *S. meliloti* infection (Figure 5). After being infected for 12 h, the expression levels of *Mt14-3-3a, Mt14-3-3b, Mt14-3-3f*, *Mt14-3-3h*, and *Mt14-3-3i* started to decline. Meanwhile, the expression levels of *Mt14-3-3d*, and *Mt14-3-3j* did not decrease until 24 h after infection. After being infected for 48 h, the expression levels of all of the *Mt14-3-3* genes showed a decline, except *Mt14-3-3e*. Thus, the expression of 9 *Mt14-3-3s* were responsive to early *S. meliloti* infection.

Additionally, we also searched the expression data of the *Mt14-3-3* genes during *S. meliloti* infection in the MtGEA database. The expression levels of the *Mt14-3-3* genes in roots did not show obvious changes after *S. meliloti* infection, with only a few showing a slight declined (Figure S3).

### Expression of *Mt14-3-3* genes after hormone treatments

The *14-3-3* genes are involved in plant hormone stress, such as BR, Me-JA, and ABA, in plants (Gampala et al., 2007; Schoonheim et al., 2007; Wang et al., 2011; Tian et al., 2015). As a model legume, *M. truncatula* can interact with rhizobia to develop nodules, which are used to help fix nitrogen. Additionally, it was reported that auxin, SA, JA, and ABA are involved in rhizobium infections and nodulation organogenesis (Yang et al., 2015). Thus, the expression levels of *Mt14-3-3* genes after IAA, ABA, SA, and JA treatments were also examined.

**Figure 6:**
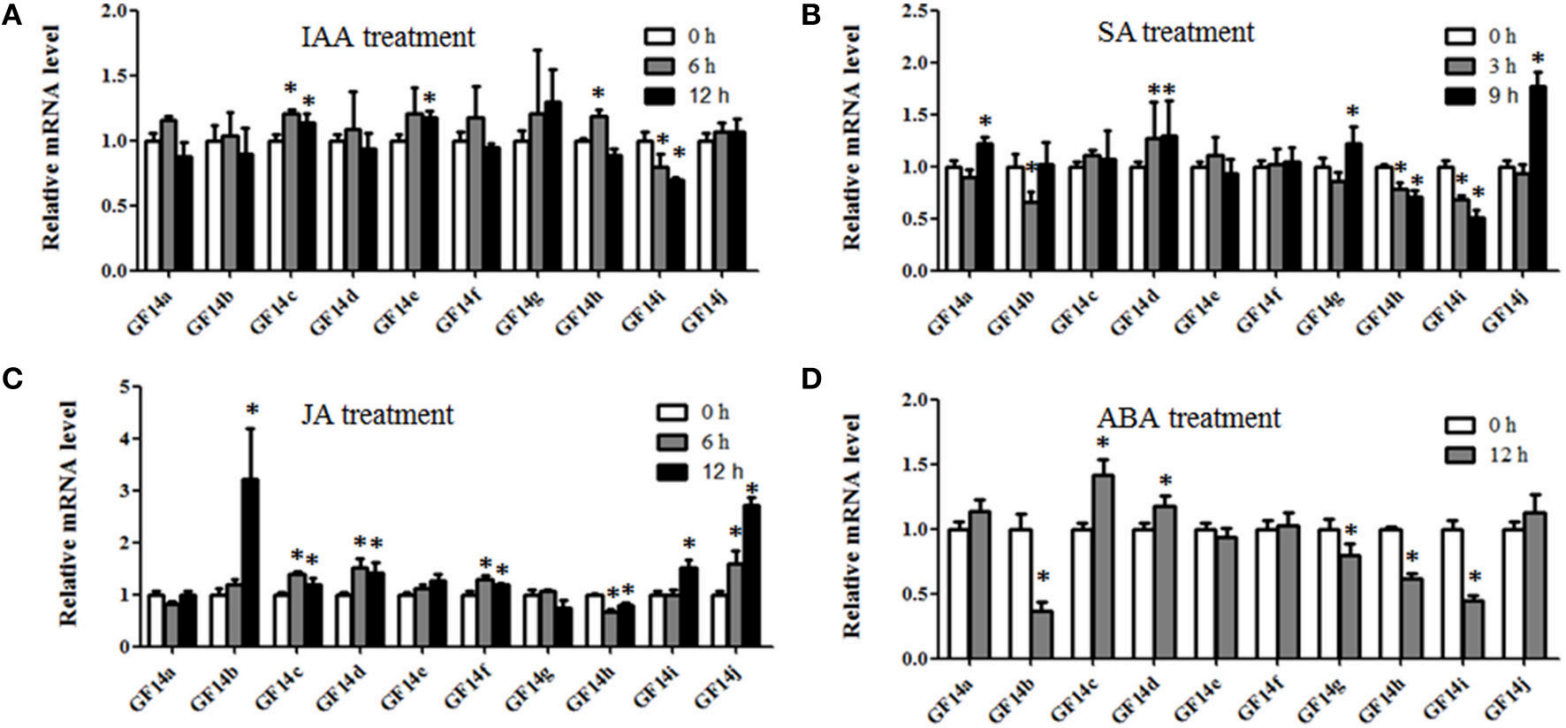
**Expression of *Mt14-3-3s* in response to hormone treatments**. The 14-day-old seedlings were treated with 1 μM IAA **(A)**, 0.5 mM SA **(B)**, 100 μM Me-JA **(C)**, and 100 μM ABA **(D)**. The significant differences between data were calculated using Student's *t* test, and indicated with an asterisks (^*^), *P* < 0.05.

After the IAA treatment, only *Mt14-3-3c, Mt14-3-3e*, and *Mt14-3-3h* were slightly induced, while *Mt14-3-3i* was reduced step by step. The remaining genes showed no obvious changes (Figure 6A). After the SA treatment, the expression level of *Mt14-3-3j* showed a significant increase, and *Mt14-3-3a, Mt14-3-3d*, and *Mt14-3-3g* were marginally induced. However, *Mt14-3-3b, Mt14-3-3h*, and *Mt14-3-3i* were slowly reduced by the SA treatment (Figure 6B). For the JA treatment, the expression levels of *Mt14-3-3c, Mt14-3-3d, Mt14-3-3f*, and *Mt14-3-3i* were induced slightly, while *Mt14-3-3b* and *Mt14-3-3j* were induced significantly, increasing almost three times after 12 h (Figure 6C). For the ABA treatment, only *Mt14-3-3c* and *Mt14-3-3d* were induced, while the expression levels of *Mt14-3-3b, Mt14-3-3g, Mt14-3-3h*, and *Mt14-3-3i* were decreased by the ABA treatment. The rest of the *Mt14-3-3* genes were almost unchanged (Figure 6D). These results showed that the expression levels of the *Mt14-3-3s* were related to these four hormones that are involved in rhizobium infection and nodulation organogenesis. Furthermore, because SA, JA, and pathogens have a close relationship, we also investigated the expression levels of *Mt14-3-3* family genes in the MtGEA database after being infected by three pathogens. This showed that there were no apparent expression changes in the *Mt14-3-3* genes after infection by *Macrophomina phaseolina*, while the expression levels were changed dramatically after *Ralstonia solanacearum* and *Phymatotrichum Root Rot* infections (Figure S4).

## Discussion

The 14-3-3 family is conserved in eukaryotes, and it forms homodimers or heterodimers, which bring different proteins into one complex. Thus, they play important roles in many plant biological progresses. Additionally, 14-3-3 families have been found and studied in many plant species, including *Arabidopsis*, rice, tomato soybean, and populus (Rosenquist et al., 2001; Jin et al., 2005; Xu and Shi, 2006; Li and Dhaubhadel, 2011; Tian et al., 2015). Knowledge of the 14-3-3 family in *M. truncatula*, an important plant species for symbiosis studies, is still limited. In the present study, we identified and analyzed the family members of Mt14-3-3, which could be used in symbiosis studies.

In this study, 10 14-3-3-family members were identified in the *M. truncatula* genome. The number of 14-3-3-family members has quite a range among different plant species. There are 13 members in *Arabidopsis*, 8 in rice, 12 in tomato, and 20 in soybean. The phylogenetic relationships of the *14-3-3* genes in these five plants species were quite close, and the phylogenetic trees showed that they were all grouped into ε and non-ε groups (Figure 3). Furthermore, a protein structure analysis showed that the 10 members of Mt14-3-3 all have nine typical antiparallel α-helices, just as 14-3-3 proteins in other species (Figure 2). These results further confirmed that the 14-3-3 family is highly conserved in eukaryotes, suggesting that the functions of Mt14-3-3s may be similar to other 14-3-3s.

We then further compared the 14-3-3s between two legume model plants, soybean and *M. truncatula*. There are 10 14-3-3 members in *M. truncatula* and 20 in soybean. The *Medicago* genome size is approximately half of the soybean genome (Young et al., 2011). The higher number of soybean 14-3-3 genes may be explained by the allotetraploid nature of soybean. But they also showed many commons between soybean and *M. truncatula*. The *14-3-3* genes of soybean and *M. truncatula* showed closer relationship in phylogenetic trees, and they were all grouped into ε and non-ε groups (Figure 3). The intron-exon pattern of the 14-3-3 family members of soybean and *M. truncatula* was found to be conserved, especially within the same phylogenetic group (Figure 1; Li and Dhaubhadel, 2011). The 14-3-3 proteins in soybean and *M. truncatula* were all composed of nine antiparallel α-helices, the amino acid sequences are highly conserved in core region except at the N-terminal and C-terminal regions (Figure 2; Li and Dhaubhadel, 2011). The conservation of the 14-3-3 family in soybean and *M. truncatula* suggested that the functions of *Mt14-3-3s* may be similar to *14-3-3s* in soybean.

To study the possible functions of *Mt14-3-3s*, the expression patterns of these genes were investigated in different tissues. The mRNA levels of these 10 *Mt14-3-3* genes can all be detected in different tissues, with quite a range (Figure 4). Some correlation was found between the expression pattern and the phylogenetic relationship results. Two sister-gene pairs, *Mt14-3-3e*/*Mt14-3-3i* and *Mt14-3-3d*/*Mt14-3-3f* (Figure 1), showed similar expression patterns (Figure 4). From the expression pattern results and the phylogenetic relationships of the *Mt14-3-3* genes (Figure 3), we speculated regarding the possible functions of certain genes in certain developmental processes. OsGF14c plays an important role in flowering in rice (Taoka et al., 2011). OsGF14c helps to assemble FT and FD proteins, which are important in flowering signaling, into one FAC complex. The expression patterns and the phylogenetic tree results indicated that *Mt14-3-3e* and *Mt14-3-3i* were the most similar Mt14-3-3 genes when compared with *OsGF14c*, but the expression level of *Mt14-3-3e* was much higher than that of *Mt14-3-3i*, indicating that *Mt14-3-3e* may play an important role in flowering. Thus, research on flowering signals in *M. truncatula* should focus on *Mt14-3-3e* and *Mt14-3-3i*, especially *Mt14-3-3e*.

As a model legume, people are interested in performing symbiosis studies in *M. truncatula*. Hormones are involved in rhizobium infection and nodulation organogenesis (Yang et al., 2015). Auxin plays a positive role, while SA, JA, and ABA play negative roles. As a result, the expression levels of *Mt14-3-3*s after treatments with these hormone ranged greatly (Figure 6), indicating that different *Mt14-3-3*s may play different roles in different developmental processes under variable hormone regulation. Rhizobial infection is the first step to develop nodules, and many genes may be involved in this early stage of rhizobial infection. In soybean, the 14-3-3s play an essential role in mature nodule development. When the expression level of the 14-3-3 homolog SGF14c/SGF14l was reduced, the nodule development and maturation in soybean was reduced, and the cytoplasm of the nodule primordia cells was degraded during nodule early development (Radwan et al., 2012). Our expression results showed that *Mt14-3-3* genes were involved in rhizobial infections. The expression levels of most of the *Mt14-3-3s* were suppressed after being infected by *S. meliloti*, except for *Mt14-3-3*c, which had an induced expression level at 12 h after the *S. meliloti* infection (Figure 5). The phylogenetic relationship results (Figure 3) showed that the *Mt14-3-3* genes most similar to *SGF14c/SGF14l* were *Mt14-3-3a, Mt14-3-3j*, and *Mt14-3-3*c. Furthermore, because the expression levels of *Mt14-3-3a* and *Mt14-3-3j* were much lower than that of *Mt14-3-3c* in *M. truncatula* (Figure 4), and that only *Mt14-3-3*c was induced after an *S. meliloti* infection, we speculated that *Mt14-3-3*c may play an essential role in the early stage of nodule formation, although more experiments are required to determine the function.

The 14-3-3 isoforms showed subcellular, tissue and functional specificity in *Arabidopsis* (Paul et al., 2005). According to our results, the Mt14-3-3 proteins also showed specificity in *M. truncatula* (Figures 4–6). According to the phylogenetic relationships of the *Mt14-3-3* genes (Figure 3), sister-gene pair, *Mt14-3-3e*/*Mt14-3-3i* (Figure 1), showed similar expression patterns in different tissues (Figure 4), but when infection by *S. meliloti*, the expression levels of *Mt14-3-3e* showed no obvious changes, while *Mt14-3-3i* showed a drastic decline in the roots. So did in hormone treatments. After the IAA treatment, *Mt14-3-3e* was slightly induced, while *Mt14-3-3i* was reduced step by step (Figure 6A). The expression level of *Mt14-3-3e* showed no obvious changes after the SA, JA and ABA treatment, while *Mt14-3-3i* were reduced by the SA, ABA treatment and induced by JA treatment (Figures 6B–D). These results showed that the isoforms, even sister-gene pairs, showed different functions, maybe that's because each isoform may interact with specific clients to carry out different function in *M. truncatula*.

Several recent high-throughput proteomic studies have been carried out to search potential 14-3-3 interactors (Chang et al., 2009; Paul et al., 2009; Jaspert et al., 2011), the results suggested that 14-3-3 may involve in many progresses, such as ion transport, hormonal signaling, pathogen, and wounding response and so on. It was also found that fundamental 14-3-3 interaction complexes are highly conserved across eukaryotes (Paul et al., 2009). Hormones, such as auxin and ABA, are involved in rhizobium infection and nodulation organogenesis (Yang et al., 2015), so the homologs of components in auxin and ABA signaling, like ARF6, ARF15, ARF18, IAA14, IAA17, IAA18, IAA19, ABF1, ABF2, ABF3, ABF4, ABI5, and AREB3, which were found to be the 14-3-3 interactors in *Arabidopsis* (Jaspert et al., 2011), may be potential 14-3-3 interactors in the symbiotic relationship in *M. truncatula. M. truncatula* is associated with N-fixing bacteria, so the components that involved in nitrogen metabolism, such as Ferredoxin-dependent Glu synthase 1 and nitrate reductase (Chang et al., 2009), may also involve in the symbiotic relationship in *M. truncatula*.

In the present study, we identified and analyzed the 14-3-3 family in *M. truncatula*. Phylogenetic and promoter analyses were performed, and gene structures and expression patterns in different tissues were studied in detail. We found that Mt14-3-3s were involved in nodule development. Although the primary functions of *Mt14-3-3*s remain largely unknown, the results in this paper lay a solid foundation for further functional investigations of the *Mt14-3-3*s.

## Author contributions

CQ and CS conceived and designed the experiments. CQ, LC, JS, YZ, HC, and DL performed the experiments. CQ and CS analyzed data. CQ, LC, and CS wrote the manuscript. All authors read and approved the manuscript.

## Funding

This work was supported by the National Natural Science Foundation of China (Grant number 31500251), the Natural Science Foundation of Zhejiang Province (Grant number LQ13C060003) and the China Scholarship Council (Grant number 201508330107).

### Conflict of interest statement

The authors declare that the research was conducted in the absence of any commercial or financial relationships that could be construed as a potential conflict of interest. The reviewer IC and the handling Editor declared a shared affiliation, and the handling Editor states that the process nevertheless met the standards of a fair and objective review.
