# Supplementary material for: Genome-Wide Identification and Expression Analysis of the 14-3-3 Family Genes in Medicago truncatula
Source: Front Plant Sci. 2016 Mar 22;7:320. doi: 10.3389/fpls.2016.00320 (PMC4801894; doi:10.3389/fpls.2016.00320)
Supplement: Supplementary file 4 [file Image1.PDF]

```
                         v10       v20       v30       v40       v50       v60       v70       v80
Medtr3g014060    ATGGGTTTTACGGTCGCAGAGAGACTAAGGAGAGAAGAGTATGTGTTCCATGCAAAACTAGCACAACAAGCGGAACGCTA
                 ATGGGTTTTACGGTCGCAGAGAGACTAAGGAGAGAAGAGTATGTGTTCCATGCAAAACTAGCACAACAAGCGGAACGCTA
Medtr0137s0020   ATGGGTTTTACGGTCGCAGAGAGACTAAGGAGAGAAGAGTATGTGTTCCATGCAAAACTAGCACAACAAGCGGAACGCTA
                         ^10       ^20       ^30       ^40       ^50       ^60       ^70       ^80
                         v90       v100      v110      v120      v130      v140      v150      v160
Medtr3g014060    CGAGGAGATGGTTTCCTTCATGCAGAAGATAGTTGTAGGGTACACACCAGCCTCAGAGCTTAGCTTGGAAGAGATGAACC
                 CGAGGAGATGGTTTCCTTCATGCAGAAGATAGTTGTAGGGTACACACCAGCCTCAGAGCTTAGCTTGGAAGAGATGAACC
Medtr0137s0020   CGAGGAGATGGTTTCCTTCATGCAGAAGATAGTTGTAGGGTACACACCAGCCTCAGAGCTTAGCTTGGAAGAGATGAACC
                         ^90       ^100      ^110      ^120      ^130      ^140      ^150      ^160
                         v170      v180      v190      v200      v210      v220      v230      v240
Medtr3g014060    TACTATCTGTCGCTTACAAGAACGCGACCGAACCACTTCGTGCGGCGTTGAGAATCCTTTCGAAGGAGGAAGAAGGTAGA
                 TACTATCTGTCGCTTACAAGAACGCGACCGAACCACTTCGTGCGGCGTTGAGAATCCTTTC AAGGAGGAAGAAGGTAGA
Medtr0137s0020   TACTATCTGTCGCTTACAAGAACGCGACCGAACCACTTCGTGCGGCGTTGAGAATCCTTTCAAAGGAGGAAGAAGGTAGA
                         ^170      ^180      ^190      ^200      ^210      ^220      ^230      ^240
                         v250      v260      v270      v280      v290      v300      v310      v320
Medtr3g014060    AAGAATGAAGATGATCACTTTGTGCATGTTAAGAAATACAAGTCCAAAGTTGAATCCGAGTTGGAGAATGTTTGTGGTAG
                 AAGAATGAAGATGATCACTTTGTGCATGTTAAGAAATACAAGTCCAAAGTTGAATC GAGTTGGAGAATGTTTGTGGTAG
Medtr0137s0020   AAGAATGAAGATGATCACTTTGTGCATGTTAAGAAATACAAGTCCAAAGTTGAATCAGAGTTGGAGAATGTTTGTGGTAG
                         ^250      ^260      ^270      ^280      ^290      ^300      ^310      ^320
                         v330      v340      v350      v360      v370      v380      v390      v400
Medtr3g014060    TATTCTGGAGTTGCTGGATTCGAAACTTATACCTTCTGCTTCATCGAGTGAGATTAGGGTTGTTTATTATCAGATGAAAG
                 TATTCTGGAGTTGCTGGATTCGAAACTTATACCTTCTGCTTCATCGAGTGAGATTAGGGTTGTTTATTATCAGATGAAAG
Medtr0137s0020   TATTCTGGAGTTGCTGGATTCGAAACTTATACCTTCTGCTTCATCGAGTGAGATTAGGGTTGTTTATTATCAGATGAAAG
                         ^330      ^340      ^350      ^360      ^370      ^380      ^390      ^400
                         v410      v420      v430      v440      v450      v460      v470      v480
Medtr3g014060    GTGATTATCAAAGGTATATGGCTGAGTTTAAGATTGGTGATGACAAAAAATCTGCTGTTGAGGATATTATCTTGTCTTAC
                 GTGATTATCAAAGGTATATGGCTGAGTTTAAGATTGGTGATGACAAAAAATCTGCTGTTGAGGATATTATCTTGTCTTAC
Medtr0137s0020   GTGATTATCAAAGGTATATGGCTGAGTTTAAGATTGGTGATGACAAAAAATCTGCTGTTGAGGATATTATCTTGTCTTAC
                         ^410      ^420      ^430      ^440      ^450      ^460      ^470      ^480
                         v490      v500      v510      v520      v530      v540      v550      v560
Medtr3g014060    AAGGCTGCACAGGATATTGCTGCGGCAGATCTTCGATCTTCTCATCCTATAAGATTGGGTTTGGCTTTGAATTTCTCGGT
                 AAGGCTGCACAGGATATTGCTGC GC GATCTTCGATCTTCTCATCCTATAAGATTGGGTTTGGCTTTGAATTTCTCGGT
Medtr0137s0020   AAGGCTGCACAGGATATTGCTGCTGCGGATCTTCGATCTTCTCATCCTATAAGATTGGGTTTGGCTTTGAATTTCTCGGT
                         ^490      ^500      ^510      ^520      ^530      ^540      ^550      ^560
                         v570      v580      v590      v600      v610      v620      v630      v640
Medtr3g014060    TTTCTATTATGAAATTCTTAACCGGTTTGACGAAGGTCTTGACATGGCTAGACAGGCTTTAGATGAAGCACGTAATGAGC
                 TTTCTATTATGAAATTCTTAACCGGTTTGACGAAGGTCTTGACATGGCTAGACAGGCTTTAGATGAAGCACGTAATGAGC
Medtr0137s0020   TTTCTATTATGAAATTCTTAACCGGTTTGACGAAGGTCTTGACATGGCTAGACAGGCTTTAGATGAAGCACGTAATGAGC
                         ^570      ^580      ^590      ^600      ^610      ^620      ^630      ^640
                         v650      v660      v670      v680      v690      v700      v710      v720
Medtr3g014060    TCAAATTGGGAGATGAATACTACAAAGACAGCACGGTTCGCATGCAACTTTTGAGAAACAACATCATCCTCTGGACTTTT
                 TCAAATTGGGAGATGAATACTACAAAGACAGCACGGTTCGCATGCAACTTTTGAGAAACAACATCA CCTCTGGACTTTT
Medtr0137s0020   TCAAATTGGGAGATGAATACTACAAAGACAGCACGGTTCGCATGCAACTTTTGAGAAACAACATCACCCTCTGGACTTTT
                         ^650      ^660      ^670      ^680      ^690      ^700      ^710      ^720
                         v730      v740      v750
Medtr3g014060    GATGACATGGACCAGCTAGACGAGCATTGA
                 GATGACA GGACCAGCTAGACGAGCATTGA
Medtr0137s0020   GATGACACGGACCAGCTAGACGAGCATTGA
                         ^730      ^740      ^750
```

Figure S1 Alignment of Medtr3g014060 and Medtr0137s0020 using the ClustalX1.81 program.
