# Supplementary material for: Genome-Wide Identification and Expression Analysis of the 14-3-3 Family Genes in Medicago truncatula
Source: Front Plant Sci. 2016 Mar 22;7:320. doi: 10.3389/fpls.2016.00320 (PMC4801894; doi:10.3389/fpls.2016.00320)
Supplement: Supplementary file 5 [file Image2.PDF]

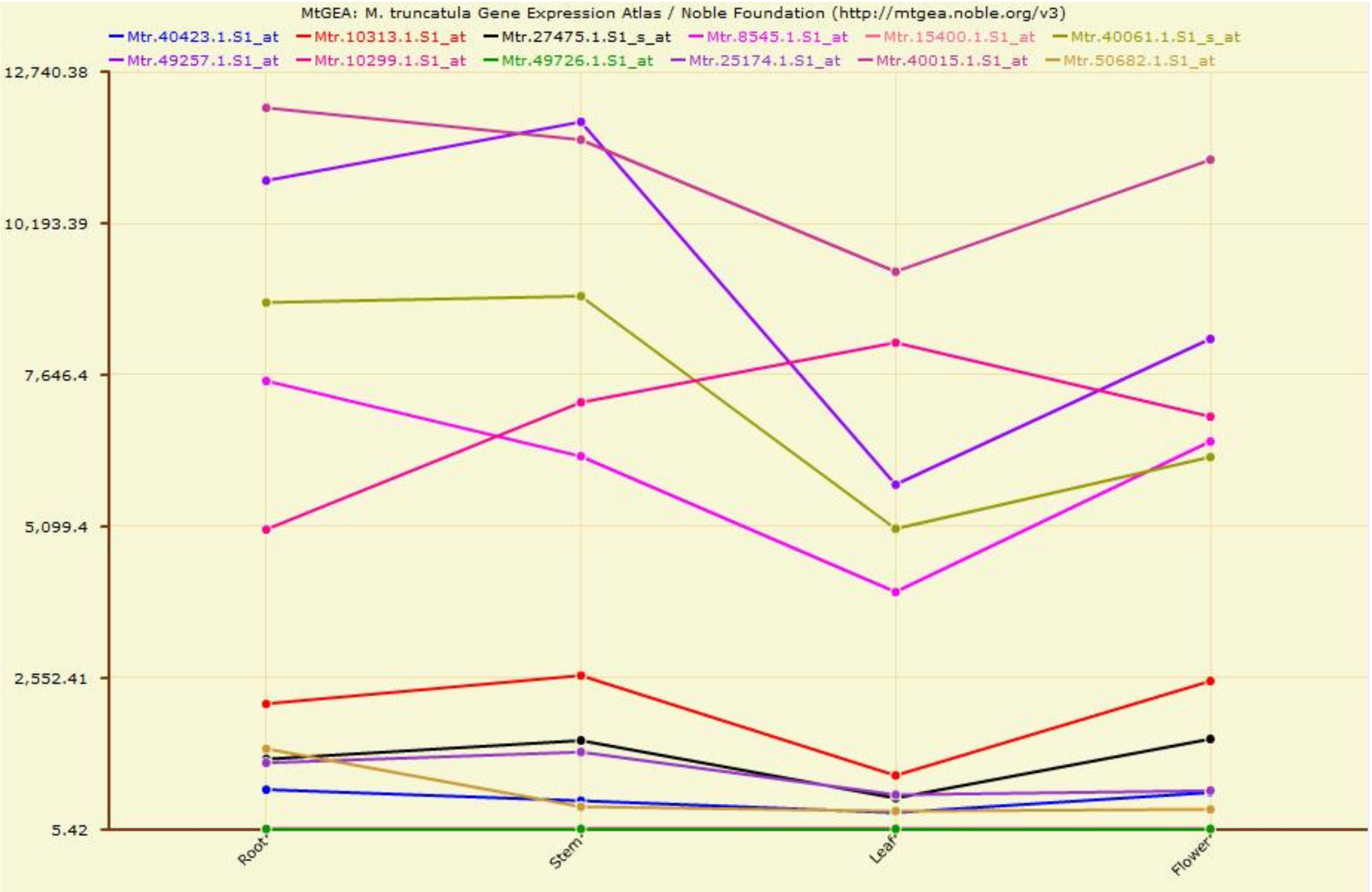

Figure S2 The tissue-specific expression patterns of Mt*14-3-3* family genes from the Medicago gene atlas (http://mtgea.noble.org/v3/). The probeset ID for each Mt*14-3-3* gene is listed in Table S2.
