# Supplementary material for: Genome-Wide Identification and Expression Analysis of the 14-3-3 Family Genes in Medicago truncatula
Source: Front Plant Sci. 2016 Mar 22;7:320. doi: 10.3389/fpls.2016.00320 (PMC4801894; doi:10.3389/fpls.2016.00320)
Supplement: Supplementary file 6 [file Image3.PDF]

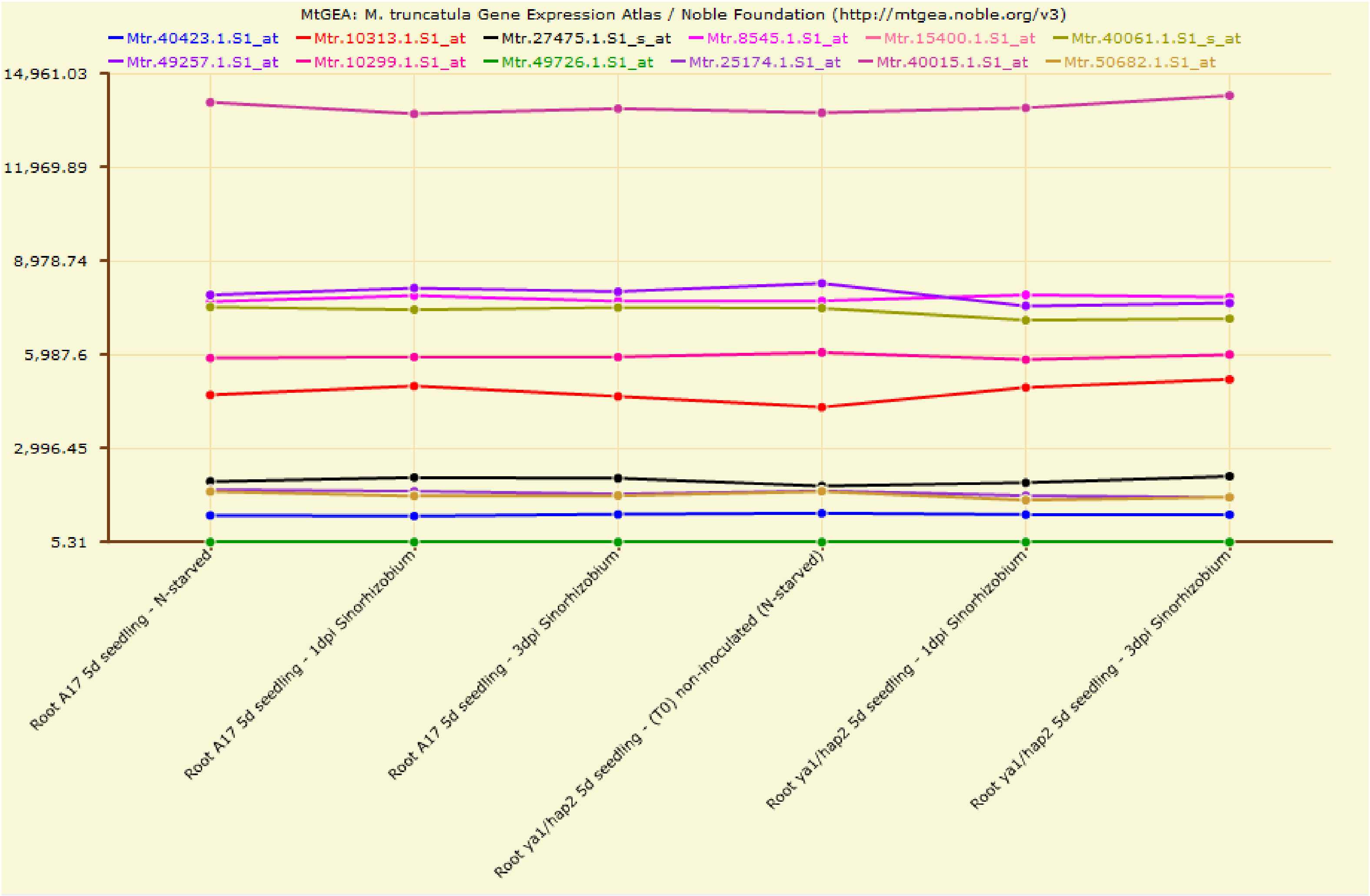

Figure S3 Expression data of Mt*14-3-3* genes after an *S. meliloti* infection from the Medicago gene atlas (http://mtgea.noble.org/v3/). The expression levels of the *Mt14-3-3* genes in roots were analyzed at different time points (1 and 3 dpi) after *S. meliloti* infection.
