# Supplementary material for: Genome-Wide Identification and Expression Analysis of the 14-3-3 Family Genes in Medicago truncatula
Source: Front Plant Sci. 2016 Mar 22;7:320. doi: 10.3389/fpls.2016.00320 (PMC4801894; doi:10.3389/fpls.2016.00320)
Supplement: Supplementary file 7 [file Image4.PDF]

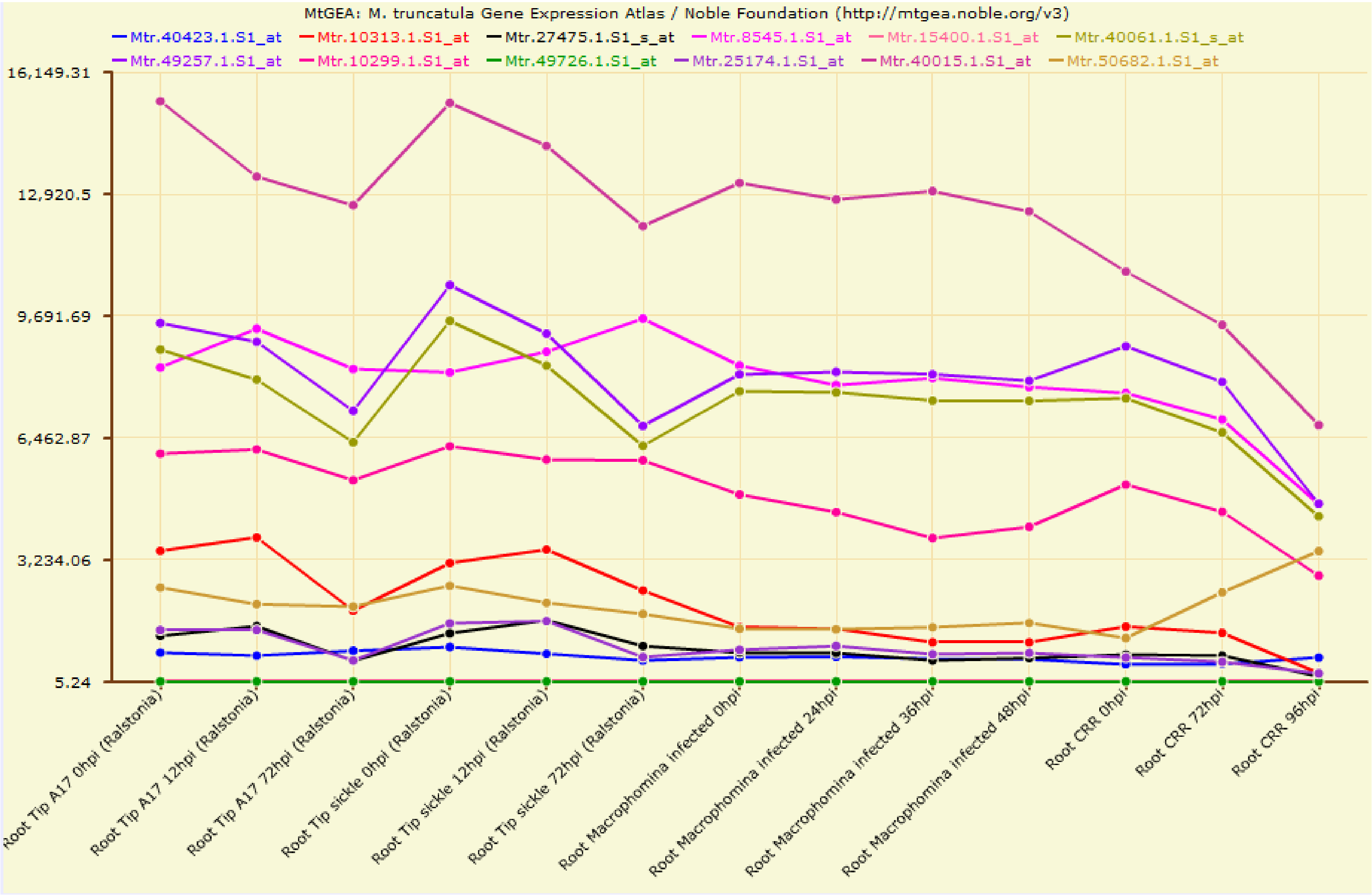

Figure S4 Expression data of Mt*14-3-3* genes during pathogen infections from the Medicago gene atlas (http://mtgea.noble.org/v3/). Ralstonia: *Ralstonia solanacearum*; Macrophomina: *Macrophomina phaseolina*; CRR: Phymatotrichum Root Rot.
